# Treatment of HR+/HER2− breast cancer in urban mainland China: results from the CancerMPact Survey 2019

**DOI:** 10.1007/s10549-022-06709-x

**Published:** 2022-08-20

**Authors:** Bhavna Murali, Laura Durbin, Sapna Vijaykumar, Linda Yang, Song Li, Linda Zhao, Stephanie Hawthorne, Gena Kanas, Christine Davis, Otávio Clark

**Affiliations:** Cerner Enviza, 2800 Rock Creek Pkwy, North Kansas City, MO 64117 USA

**Keywords:** Breast cancer, Chemotherapy, Treatment patterns, Cancer treatment, China

## Abstract

**Purpose:**

To report the treatment utilization patterns for hormone receptor-positive (HR+)/human epidermal growth factor receptor 2-negative (HER2−) breast cancer in urban mainland China (CancerMPact®).

**Methods:**

The results presented are from an online survey conducted in September 2019 with 45 physicians treating breast cancer patients from 11 cities in mainland China.

**Results:**

Surveyed physicians reported that Stage I HR+/HER2(−) breast cancer patients are often treated with surgery alone (42%), whereas the use of surgery in combination with systemic therapy with or without radiotherapy increases in later stages (Stage II 67%, Stage III 77%). Doxorubicin–cyclophosphamide (AC)-based regimens were the most common in both the neoadjuvant and adjuvant settings in HR+/HER2(−) breast cancer patients, across all stages. In metastatic patients, use of surgery and radiotherapy decreases in favor of utilization of systemic therapy alone. Pre- and post-menopausal metastatic patients were frequently treated with hormone therapy or AC-based regimens in first line. Regardless of the first-line therapy administered, capecitabine-based regimens were commonly used in second line. In third line, chemotherapy regimens containing capecitabine or gemcitabine were given to nearly 40% of HR+/HER2(−) breast cancer patients. There were no standard of care regimens established for fourth or greater lines of treatment. In metastatic HR+/HER2(−) breast cancer, physicians reported 50% objective response rates in first-line settings with a progression-free survival of 16 months.

**Conclusion:**

HR+/HER2(−) breast cancer patients in urban mainland China were prescribed chemotherapy regimens more frequently than CDK4/6 inhibitors. Treatment practices varied, with physicians reporting the use of multiple modalities and treatment regimens for their patients.

**Supplementary Information:**

The online version contains supplementary material available at 10.1007/s10549-022-06709-x.

## Introduction

Breast cancer is the leading cause of cancer-related deaths in women worldwide [1]. The incidence of breast cancer has been decreasing in the United States (USA) and Western Europe (EU5), while in Asian countries, including China, breast cancer incidence and mortality have been rising over the past several years [2]. In China, it is estimated that 307,184 people were diagnosed with breast cancer in 2019 [3]. Lifestyle changes, obesity, and lack of access to reliable diagnostics and effective therapies have contributed to this rise in breast cancer mortality in China and other developing countries [4].

Aberrations in hormone receptor (HR) and human epidermal growth factor receptor 2 (HER2) pathways are frequently observed in breast cancer patients [5]. Based on molecular biomarkers, breast cancer can be classified into at least four different subtypes, with different treatments and prognoses: HR-negative and HER2-enriched, HR-positive and HER2-negative luminal A, HR-positive and HER2-positive luminal B and triple negative/basal-like, which is a subtype that is negative for both HR and HER2. In 2019, approximately 54% of breast cancer cases in China were diagnosed with HR+/HER2(−), 17% with HR+/HER2+, 12% with HR(−)/HER2+ , and 17% with triple negative subtypes [3]. The HR+/HER2(−) subtype is likely to remain the most commonly diagnosed type of breast cancer, and its annual incidence is predicted to increase by 2% over the next 3 years [3, 6–8].

Currently available treatment options for breast cancer include surgery, radiation, and systemic therapies, including chemotherapy, hormone therapy, immunotherapies, and targeted therapies. Choice of modality is highly complex and is based on the stage at diagnosis, biomarker status, subtype, patient age, comorbidities, and menopausal status [9]. Over the last few decades, treatment options for metastatic HR+/HER2(−) breast cancer patients have evolved with the approval of targeted agents. Hormone therapy is still widely used as a standard of care either as single agent or in combination with other systemic drugs such as chemotherapy and CDK4/6 inhibitors [10, 11]. Most of the studies that determined the effectiveness of these treatments were performed in western countries and some Asian institutions have questioned the generalizability of these data to Eastern women, underlining the need to better understand how these patients are treated in Asia, particularly in China [12].

The current patterns of care of breast cancer patients in Asia, and in China specifically, are largely unknown [12]. A better understanding of treatment practices could highlight the existing strengths and knowledge gaps and accordingly inform clinical trial design and resource allocations in the local healthcare systems.

Previously, we reported real-world evidence (RWE)-based data collected from various registries and primary physician surveys that identified how physicians treat non-small-cell lung cancer and melanoma in the USA and EU5 countries and provided a better understanding of the treatment dynamics in various markets [13, 14]. Here, we aim to report the results of a survey of physicians treating HR+/HER2(−) breast cancer patients in urban mainland China.

## Materials and methods

CancerMPact® (CMP) is a proprietary database from Cerner Enviza (formerly known as Kantar Health), which contains oncology epidemiology and cancer treatment data [3]. Cerner Enviza conducts annual surveys with physician specialists, including oncologists, treating cancer patients across various tumor types in the USA, EU5, Japan, and China. In 2019, this CMP study surveyed 4859 physicians from these geographic regions about the treatment of 31 different tumors.

The survey performed in China for breast cancer in 2019 recruited 45 physicians from 11 cities in mainland China using an online format. To be eligible for the survey, physicians must have been a board-certified practitioner of one of the following specialties: medical oncology, surgical oncology, or surgery; be in practice for a minimum of 5 years; and must have treated a minimum of 23 breast cancer patients per month.

The survey questionnaire was developed by an internal Oncology team, who reviewed current treatment algorithms recommended by international guidelines, such as those from the Chinese Society of Clinical Oncology (CSCO) [15], as well as new drug approvals by China’s national agency for regulating drugs and medical devices, The National Medical Products Administration (NMPA). This was supplemented with English and Chinese language literature reviews of registrational clinical trial data from medical journals and oncology conferences to identify the practice behavior and potential changes in the treatment of breast cancer in China.

The 2019 breast cancer survey asked physicians about their oncology practice experience, their patient characteristics (e.g., patient volume, the stages of patients seen and patient biomarker status) and to consider patients that they had treated in the last 6 months. Detailed questions covered aspects of treatment by stage and subtype, and physicians were asked to report the proportion of their patients treated by each modality type and the percentage of patients treated with each systemic therapy regimen, the duration of the treatment, and their results. Chemotherapy agents, subtype-specific targeted agents, and hormonal agents at each line of therapy were also reported. Physicians were asked to report treatment outcomes (percentage of recurrence and patient prognosis) at each line of therapy up to fifth line, if applicable. The survey explicitly communicated to physicians that they should answer questions on their utilization of Western treatment modalities and Western medicines and to not include Traditional Chinese Medicine (TCM) in their responses. In addition, definitions of relapse and refractory patients, and endpoints, including overall response rate (ORR) or progression-free survival (PFS), were not provided in the survey and were based on the physicians' own clinical practice and experience.

The online physician questionnaire was programmed, fielded, and hosted by an online-survey company and the anonymized raw data were securely transferred to Cerner Enviza, where data analysis was completed. Data from the survey are reported as unweighted averages of all responses and no formal statistical treatment was applied to the results.

The survey was fielded in September 2019. The CMP data source used for this research does not collect or use patient-level data, or any data involving people, medical records, or human samples. The researchers did not review patient charts, survey patients, or interact with patients in any way. All information is retrieved from online physician surveys regarding information around overall treatment patterns. Therefore, no Institutional Review Board approval was necessary.

## Results

On average, the 45 Chinese physicians included in this survey had 16.3 years of medical experience post-residency training and treated 41 breast cancer patients per month. More than half of them were medical oncologists from Level III hospitals and together they treated 1848 breast cancer patients per month (Table 1). Over half of the respondents (60.0%) were located in Beijing, Shanghai, or Guangzhou/Shenzhen.

**Table 1:** Characteristics of physicians surveyed, China, 2019

Characteristics of physician respondents	*N*/%
Number of physicians surveyed	45
Average number of years of practice after residency (range)	16.3 (5–30)
Average number of breast cancer patients treated by each physician monthly (range)	41.1 (23–100)
Board-certified specialty	
Medical oncology (%)	57.8%
Surgical oncology (%)	22.2%
Surgery (%)	20.0%
Hospital levels	
Level III (%)	91.1%
Level II (%)	4.4%
Cancer specialty (%)	4.4%
Practice locations	
Beijing (%)	22.2%
Shanghai (%)	22.2%
Guangzhou/Shenzhen (%)	15.6%
Xi’an (%)	6.7%
Chengdu (%)	6.7%
Tianjin (%)	6.7%
Other^a^ (%)	20.0%

^a^Locations not individually reported if less than 5%

### Treatment modalities and regimens in early-stage HR+/HER2(−) breast cancer

According to the physician respondents, 42% of their stage I patients received surgery only, while almost half received a combination of surgery with radiation and/or systemic therapy. As the disease progresses, the triple combination of surgery, radiation, and systemic therapy is increasingly utilized due to the higher tumor burden associated with more advanced disease (Table 2).

**Table 2:** Initial treatment modalities for HR+/HER2(−) breast cancer patients, Stages I–III, China, 2019

Modality	Stage I (%)	Stage II (%)	Stage III (%)
Surgery only	41.9	14.1	4.5
Surgery, systemic therapy	26.5	35.4	36.4
Surgery, RT, systemic therapy	16.1	31.9	41.0
Surgery, RT	4.9	3.5	2.2
Systemic therapy only	4.4	4.6	6.4
No therapy/observation^a^	2.6	1.8	3.3
RT, systemic therapy	2.4	7.6	5.5
RT only	1.2	1.1	1.7

Systemic therapy includes chemotherapy, biologic therapy as well as HER2 targeted and other targeted agents. Survey of 45 physicians who treat a total of 1,848 breast cancer patients monthly, conducted in September 2019; 36 physicians completed data for stage I, 43 physicians completed data for stage II, and 45 physicians completed data for stage III. Survey of 45 physicians who treat a total of 1,848 breast cancer patients monthly, conducted in September 2019

*RT* radiation therapy

^a^In the survey, supportive care and/or traditional Chinese medicine are included within no therapy/observation

In both the neoadjuvant (Table 3) and adjuvant (Table 4) settings, and across stages I–III, physicians reported using doxorubicin in combination with cyclophosphamide (AC) with or without a taxane in more than 40% of their HR+/HER2(−) breast cancer patients. Regimens with an epirubicin and cyclophosphamide (EC) backbone are also quite commonly used in early-stage patients in the perioperative setting (Tables 3, 4).

**Table 3:** Utilization of neoadjuvant systemic therapy regimens in HR+ /HER2− breast cancer patients, Stages I–III, China, 2019

Regimen	Stage I		Stage II		Stage III	
	Utilization (%)	Average number of months (range)	Utilization	Average number of months (range)	Utilization	Average number of months (range)
AC, docetaxel	19.0	5.0 (3–7)	22.2%	4.5 (2–8)	22.7%	4.7 (2–8)
AC, paclitaxel	14.1	5.7 (3–8)	6.4%	4.8 (3–6)	9.1%	4.2 (3–6)
EC	11.6	5.3 (2–8)	^a^	–	^a^	–
Other	11.8	6.0	11.2%	5.1	13.2%	5.6
Other AC-based	12.2	5.6	17.0%	4.4	20.7%	4.6
Other docetaxel-based	6.8	9.0	14.4%	5.6	11.5%	5.7
Other EC-based	9.9	9.0	11.2%	5.8	13.0%	5.6
Other epirubicin-based	14.5	7.6	13.3%	5.2	6.7%	5.9

Seventeen physicians completed data for stage I, 37 physicians completed data for stage II, and 44 physicians completed data for stage III. “Other” category includes various therapies used in < 5% of patients each

*AC* doxorubicin, cyclophosphamide, *EC* epirubicin, cyclophosphamide

^a^Less than 5%

**Table 4:** Utilization of adjuvant systemic therapy regimens in HR+ /HER2− breast cancer patients, Stages I–III, China, 2019

Regimen	Stage I		Stage II		Stage III	
	Utilization	Average number of months (range)	Utilization (%)	Average number of months (range)	Utilization (%)	Average number of months (range)
AC	13.8%	4.3 (1–6)	10.1	5.4 (3–8)	9.8	5.5 (4–6)
AC, docetaxel	14.6%	5.7 (3–8)	12.9	5.5 (3–6)	19.3	5.5 (3–8)
EC, docetaxel	9.6%	5.4 (4–6)	11.2	5.4 (3–8)	8.5	5.8 (4–8)
Other doxorubicin-based	^a^	–	5.5	4.8 (2–6)	5.3	6.4 (5–13)
Other AC-based	21.1%	5.0	28.7	5.8	26.2	5.5
Other EC-based	17.7%	10.5	15.2	5.4	15.1	5.5
Other	19.1%	9.4	16.4	6.1	15.8	5.7

Twenty-eight physicians completed data for Stage I, 38 physicians completed data for Stage II, and 43 physicians completed data for Stage III. Other” category includes various therapies used in < 5% of patients each

*AC* doxorubicin, cyclophosphamide, *EC* epirubicin, cyclophosphamide

^a^Less than 5%

According to the physicians, approximately half of the stage I patients remained in remission for at least a decade (Table 5). As disease progresses, the proportion of patients in remission starts to drop with only 17% of stage III patients not experiencing relapse within 10 years of initial therapy. Across stages I–III, approximately 20% of the patients remained disease-free between 6 and 10 years following therapy. The proportion of patients with refractory tumors or disease relapse within a year, varied from 12% for stage I to 32% for stage III (Table 5).

**Table 5:** Recurrence patterns in stage I–III HR+ /HER2− breast cancer patients in China after receiving initial treatment, China, 2019

Regimen	Stage I (%)	Stage II (%)	Stage III (%)
Patients who do not respond to therapy (refractory)	3.4	5.8	11.5
Patients who respond but relapse within 1 year of therapy	8.9	13.2	20.4
Patients who respond but relapse between 1 and 5 years of therapy	17.9	25.1	30.5
Patients who respond but relapse between 6 and 10 years of therapy	22.0	22.9	20.2
Patients who do not relapse within 10 years of therapy	47.8	32.9	17.2

Survey of 45 physicians who treat a total of 1,848 breast cancer patients monthly, conducted in September 2019; 36 physicians completed data for Stage I, 43 physicians completed data for Stage II, and 45 physicians completed data for Stage III

In nearly 60% of the patients with stage I breast cancer with local recurrence, surgery, either alone or in combination with systemic therapy, was the modality of choice for managing local recurrence (Table 6). Systemic therapy alone was used in 9% to 12% of stage I–III breast cancer patients with local recurrence (Table 6). The proportion of patients with local recurrence requiring a combination of surgery, radiation, and systemic therapy increased from 18% in stage I to 34% in stage III breast cancer (Table 6).

**Table 6:** Stage specific treatment modality utilization in stage I–III HR+ /HER2− breast cancer patients who had local recurrent disease, China, 2019

Modality	Stage I (%)	Stage II (%)	Stage III (%)
No therapy/observation	1.7	2.7	3.4
Surgery only	30.9	13.9	8.3
Systemic therapy only	9.0	9.6	12.2
RT only	1.4	1.5	1.4
Surgery, systemic therapy	29.3	33.6	27.9
Surgery, RT	4.8	4.3	3.1
Systemic therapy, RT	4.7	6.9	9.5
Surgery, RT, systemic therapy	18.3	27.5	34.2

Systemic therapy includes chemotherapy, hormone therapy, as well as biologic therapy and targeted therapies. 35 physicians completed data for Stage I, 43 physicians completed data for Stages II and 45 physicians completed data for Stage III

*RT* radiation therapy

### Treatment regimens used in advanced HR+/HER2(−) breast cancer

In stage IV HR+/HER2(−) breast cancer, systemic therapy is the mainstay of treatment. Physicians reported that over 80% of patients receive systemic therapy in their first-line treatment, either alone or in combination with other modalities (Table 7). About 40% of metastatic patients also receive surgery as part of a multimodal treatment approach, often in combination with systemic therapy and/or radiation. Although surgery is not associated with improvement in survival in metastatic patients [16, 17], clinical guidelines recognize that it may still be suitable in some patients to achieve local control of the primary tumor [18]. In pre-menopausal stage IV patients, AC-based regimens or hormone therapies were frequently used, with each group having about 25% utilization share (Table 8). Docetaxel was the most frequently prescribed taxane in these advanced HR+/HER2(−) breast cancer patients. Palbociclib, the only CDK4/6-targeted agent approved in China at the time the survey, was conducted and was prescribed in 5% of metastatic patients, regardless of menopausal status (Table 8). The general treatment pattern of post-menopausal patients is similar to that of pre-menopausal patients, with AC-based regimens and hormone-alone therapies getting highest utilization. Aromatase inhibitors were most frequently prescribed in post-menopausal patients while pre-menopausal patients were given tamoxifen. Everolimus was used in 10% of post-menopausal patients while it was rarely given to pre-menopausal patients (Table 8).

**Table 7:** First-line treatment modalities for metastatic HR+/HER2(−) breast cancer patients, China, 2019

Modality	Stage IV (%)
No therapy/observation	6.5
Systemic therapy only	28.2
Systemic therapy, RT	20.6
Surgery, RT, systemic therapy	19.8
Surgery, systemic therapy	14.6
Surgery only	3.9
Surgery, RT	3.6
RT only	2.9

Systemic therapy includes chemotherapy, hormone therapy, as well as biologic therapy and targeted therapies. 42 physicians completed data for Stage IV patients

*RT* radiation therapy

**Table 8:** Utilization of first-line systemic regimens in pre-menopausal and post-menopausal patients with HR+/HER2− breast cancer, China, 2019

Regimen	Pre-menopausal		Post-menopausal	
	Utilization	Average number of months (range)	Utilization	Average number of months (range)
Bevacizumab-based	7.2%	6.3 (3–12)	7.6%	6.1 (3–12)
Carboplatin-based	5.6%	5.2 (3–6)	^a^	–
AC	8.2%	4.6 (3–6)	7.3%	4.7 (1–6)
AC, docetaxel	8.0%	5.7 (3–12)	7.4%	5.8 (3–12)
Docetaxel, capecitabine	6.2%	5.8 (4–8)	^a^	–
Everolimus-based	^a^	–	9.6%	8.5
Tamoxifen	13.6%	11.4 (1–60)	^a^	–
Palbociclib, hormone therapy	4.8%	11.2 (6–24)	4.6%	17.3 (3–60)
Fulvestrant	6.1%	7.6 (1–14)	7.3%	12.1 (3–24)
Aromatase inhibitor	6.3%	9.6 (1–60)	16.7%	12.0 (1–36)
Other	18.3%	5.7	16.2%	5.2
Other capecitabine-based	4.8%	6.8	6.9%	7.1
Other AC-based	8.1%	6.2	8.3%	6.1

Forty-two physicians completed data for pre- and post-menopausal patients. “Other” category includes various therapies used in < 5% of patients each

*AC* doxorubicin, cyclophosphamide

^a^Less than 5% utilization

Second-line treatment choice is often influenced by exposure to prior therapy. Combination regimens containing bevacizumab were used in about 20% of HR+/HER2(−) breast cancer patients who had previously received everolimus with anti-hormonal therapy, while about 8% received palbociclib hormonal therapy and 20% continued with everolimus-based hormonal therapy (Table 9). Patients who progressed on single-agent non-steroidal hormonal therapy in front-line settings were frequently prescribed hormonal therapy-based combinations (15%) or hormone therapy as single agent (23%) (Table 9). Chemotherapy combination regimens with capecitabine or gemcitabine were commonly used in second line following progression after single-agent aromatase inhibitors (Table 9). Palbociclib was used in less than 5% of the patients in second line if they progressed on a single-agent aromatase inhibitor (Table 9). Once the disease progressed, about 25% of the patients who received palbociclib with a hormonal agent in first line continued to receive palbociclib-based regimens in second line, but most other patients receive chemotherapy-based regimens (Table 9). Capecitabine-based treatments were reported in about 34% of the patients after progressing on first-line palbociclib hormonal therapy combination (Table 9).

**Table 9:** Second-line utilization by systemic regimen according to first-line regimen received in metastatic HR+/HER2− breast cancer, China, 2019

Regimen	Received Everolimus plus hormone agent 1st line (%)	Received non-steroidal aromatase inhibitor alone 1st line (%)	Received palbociclib plus hormone agent 1st line (%)
Hormone therapy	9	23	1
Bevacizumab-based	19	11	5
Everolimus, hormone therapy	20	12	12
Palbociclib, hormone therapy	8	3	25
Nab-paclitaxel	2	5	5
Chemotherapy, hormone therapy	5	0	2
Gemcitabine-based	9	8	5
Capecitabine-based	17	21	34
Doxorubicin/cyclophosphamide-based	8	14	9
Other	4	3	3

Survey of 45 physicians who treat a total of 1,848 breast cancer patients monthly, conducted in September 2019; 17 physicians completed data for Everolimus plus hormone, 24 physicians completed data for non-steroidal aromatase inhibitor alone, and 11 physicians completed data for palbociclib plus hormone. “Other” category includes various therapies used in < 5% of patients each

In the third-line setting, chemotherapy containing capecitabine (25%) or gemcitabine (17%) was most commonly used to treat metastatic HR+/HER2(−) breast cancer patients (Table 10). Nearly one-third of the patients were treated with hormone therapy, either as a single agent (13%) or in combination with everolimus (13%) or palbociclib (5%; Table 10).

**Table 10:** Third-line utilization by systemic regimen received in HR+/HER2− breast cancer, China, 2019

Modality	Third line	
	Utilization (%)	Average number of months (range)
Gemcitabine-based	16.9	5.7 (2–12)
Capecitabine-based	14.6	4.8 (2–12)
Everolimus, hormone therapy	13.5	7.3 (3–12)
Hormone therapy	12.8	5.9 (3–12)
Bevacizumab-based	10.7	5.9 (2–12)
Capecitabine	9.1	5.6 (3–12)
Palbociclib, hormone therapy	5.3	8.4 (5–12)
AC-based	5.2	5.8 (4–12)
Other	12.0	4.8

Survey of 45 physicians who treat a total of 1848 breast cancer patients monthly, conducted in September 2019; 32 physicians completed data for this question. Other” category includes various therapies used in < 5% of patients each

*AC* doxorubicin, cyclophosphamide

In both fourth and fifth lines, the most commonly used therapy was anti-endocrine. More than 30% of fourth-line patients and over 40% of fifth-line patients received an anti-endocrine therapy either alone or in combination with either palbociclib or everolimus (Table 11). Capecitabine-based regimens were prescribed in over 20% of fourth-line cases (Table 11). As available treatment options diminish in later lines, reliance on clinical trials increases. About 6% of fourth-line patients were participants in clinical trials while 13% enrolled in clinical trial studies in fifth-line settings.

**Table 11:** Utilization of fourth-line and fifth-line systemic regimens in HR+/HER2− breast cancer, China, 2019

Regimen	Fourth line		Fifth line	
	Utilization (%)	Average number of months (range)	Utilization (%)	Average number of months (range)
Hormone therapy alone	15.6	5.1 (2–12)	24.0	5.1 (2–12)
Everolimus, hormone therapy	10.0	7.2 (3–12)	11.6	6.7 (3–12)
Palbociclib, hormone therapy	10.2	6.9 (3–12)	6.1	7.1 (3–12)
Nab-paclitaxel	6.9	4.0 (3–6)	6.1	4.1 (3–6)
Capecitabine	5.7	3.8 (3–6)	7.8	4.0 (2–6)
Bevacizumab-based	8.6	4.9 (3–6)	3.2	7.8 (4–12)
Gemcitabine-based	5.5	5.1 (3–12)	7.8	6.0 (3–12)
Investigational drug (clinical trial)	6.1	–	13.3	–
Other capecitabine-based	16.7	4.6	7.7	5.8
Other	14.7	4.1	12.4	3.7

Survey of 45 physicians who treat a total of 1,848 breast cancer patients monthly, conducted in September 2019; 22 physicians completed data for fourth line and 18 physicians completed data for fifth line. “Other” category includes various therapies used in < 5% of patients each

### Treatment outcomes in advanced breast cancer

Nearly 60% of pre- and post-menopausal HR+/HER2(−) breast cancer patients experience disease progression following first-line therapy. Of these, about 40% of patients will receive therapy in second line, and rates of subsequent therapy decrease for later lines, with less than 15% of patients receiving sixth line upon progression (Table 12). About 20% of first-line patients do achieve remission; however, remission rates significantly decrease, to less than 10% in fourth line and beyond (Table 12).

**Table 12:** Physician-reported outcomes of metastatic breast cancer patients who received later lines of systemic therapy in HR+/HER2− breast cancer, China, 2019

Outcomes	First line to second line		Second line to third line (%)	Third line to fourth line (%)	Fourth line to fifth line (%)	Fifth line to sixth line (%)
	Pre-menopausal (%)	Post-menopausal (%)				
Patients who achieved a long-term response and never received the next line of systemic therapy	20.4	22.0	18.8	10.5	9.3	8.5
Patients who did not achieve a long-term response and who died before receiving the next line of therapy	17.0	16.8	25.9	43.2	48.2	49.6
Patients whose disease progressed and who are alive but did not receive the next line of systemic therapy (due to patient's choice, comorbidities, age, costs, etc.)	20.4	22.1	26.4	25.1	26.0	28.1
Patients whose disease progressed and who received the next line systemic therapy	42.2	39.2	28.9	21.2	16.5	13.8

Forty-two physicians completed data for first to second line (pre-menopausal), 42 physicians completed data for first to second line (post-menopausal), 41 physicians completed data for second to third line, 33 physicians completed data for third to fourth line, 24 physicians completed data for fourth to fifth line, 19 physicians completed data for fifth to sixth line

Based on their clinical experience and practice, physicians estimated just over 50% disease regression in first line, with patients not progressing to second line for about 16 months; but clinical benefit from treatment decreased with each line of therapy (Table 13). Regardless of line of therapy, over 20% of the patients exhibited stable disease with the respective administered systemic therapy (Table 13).

**Table 13:** Physician-reported outcomes of various lines of systemic therapies in HR+/HER2− breast cancer patients, China, 2019

Modality	CR (%)	PR (%)	SD (%)	Average PFS (mos)
First line	20.8	29.6	27.2	16.3
Second line	13.6	27.7	25.9	11.1
Third line	5.2	20.1	27.8	7.0
Fourth line	3.8	15.4	26.7	5.2
Fifth line	2.9	11.2	22.0	4.3

Survey of 45 physicians who treat a total of 1,848 breast cancer patients monthly, conducted in September 2019; For response rates, 42 physicians completed data for first line, 41 physicians completed data for second line, 33 physicians completed data for third line, 24 physicians completed data for fourth line, and 19 physicians completed data for fifth line; For PFS: 40 physicians completed data for first line, 39 physicians completed data for second line, 32 physicians completed data for third line, 23 physicians completed data for fourth line, and 18 physicians completed data for fifth line.

*CR* complete response rate; *PR* partial response rate; *SD* stable disease; *PFS* progression-free survival

## Discussion

Despite recent advances achieved in early diagnosis and targeted therapies, breast cancer continues to be the leading cause of cancer-related deaths worldwide. These cancer patients respond well to hormonal therapies; however, many will have disease progression after just over a year on hormonal monotherapy [9, 10].

In early stages of breast cancer, surgery is commonly used along with an adjuvant therapy involving systemic agents or radiation. HR+ breast cancer patients respond well to hormonal therapy, which can be either aromatase inhibitors or estrogen receptor binders/degraders. CSCO guidelines emphasize the importance of hormonal therapy, but also state that some patients, such as those with large (> 2 cm) tumors or nodal involvement, may be suitable for treatment with adjuvant chemotherapy. In such cases, hormonal therapy is often administered sequentially after adjuvant chemotherapy [15].

Our research revealed a similar utilization pattern of systemic therapies in both neoadjuvant and adjuvant settings in urban mainland China and elsewhere globally for treating HR+/HER2(−) breast cancer patients [3]. However, the choice of chemotherapy treatment regimen may differ slightly across various markets. In urban mainland China, physicians did show a preference for AC plus taxane regimens, over docetaxel plus cyclophosphamide, which are more commonly used in the US, whereas EC-based regimens are dominant among EU5 physicians for treating stage I HR+/HER2(−) breast cancer patients.

International guidelines for the treatment of metastatic HR+/HER2(−) breast cancer recommend chemotherapy or hormone therapy as the first therapeutic choice for most patients, either as a single agent or in combination with a CDK4/6 inhibitor [9, 10]. The optimal sequence for using hormonal therapy alone or in combination with targeted therapy in front-line settings in metastatic patients is not strictly defined and is often based on patient characteristics. We noted in our survey that, in urban mainland China, physicians most commonly treat pre-menopausal stage IV patients with front-line chemotherapy combination regimens.

Palbociclib was approved in China in July 2018 and launched on September 2018 [19]. At the time that this survey was conducted (September 2019), palbociclib was the only CDK4/6 inhibitor approved by NMPA and its usage in front line was about 5%, which is at a much lower proportion than in the USA, where CDK4/6 plus aromatase inhibitors have become the standard of care for treating HR+/HER2(−) metastatic patients regardless of menopausal phase [3]. In clinical studies, palbociclib with letrozole was shown to provide significant progression-free survival over letrozole alone in HR+/HER2(−) metastatic breast cancer patients [20]. Since the time of survey fielding, there has been a flurry of development in this space, including the approval of two CDK4/6 inhibitors. Abemaciclib was first approved in December 2020 for the treatment of metastatic patients [21], and most recently, in January 2022, it also received approval for use in the adjuvant setting [22]. In addition, in December 2021, a domestically developed CDK4/6 inhibitor, dalpiciclib, was approved by the NMPA [23]. Around the end of 2020, the first generic palbociclib, also got approved, but is not set to launch until the branded agent’s patent expiration in January 2023. A fourth CDK4/6 inhibitor, ribociclib, was reported to provide significant improvements in overall survival when combined with fulvestrant relative to fulvestrant plus placebo in HR+/HER2(−) metastatic breast cancer patients [24]. However, ribociclib is not yet on the market in China; an NDA is under regulatory review by the NMPA and the approval may not be too far in future.

Overall, this influx of several CDK4/6 inhibitor options could push adoption in favor of this class of agent in first-line patients. However, the significantly higher cost of these targeted agents, compared to chemotherapies and hormone therapies, has the potential to impact uptake by physicians. In China, approvals may not translate to clinical use unless the drug is made affordable to patients by inclusion in the National Reimbursement Drug List (NRDL), which provides up to 80% reimbursement of drug cost to patients. Nearly 1 year after approval, abemaciclib has finally entered the NRDL in January 2022, making it a more affordable CDK4/6 inhibitor option to patients than palbociclib. Despite palbociclib being the first-in-class to enter the Chinese market, it is yet to be included in the NRDL. The branded agent did, however, drop in price by 54% in January 2021, shortly following the approval of the first palbociclib generic, in an attempt to garner some utilization in this competitive market.

Upon disease progression, fulvestrant, a selective estrogen receptor degrader (SERD), is the hormone agent of choice for second and subsequent lines of therapies in the USA and EU5 [3]. In urban mainland China, there is no defined algorithm, with docetaxel-based chemotherapy alone or with EC for second line (data not shown) and capecitabine or gemcitabine-based chemotherapy for third line identified as the most commonly utilized options among physicians surveyed (Table 10). For fourth and subsequent lines, physicians in China did not report a specific standard of care and often included multiple therapies including hormone therapy alone, chemotherapies, or targeted therapies. With large patient numbers receiving the next line of therapy, current treatment options are tolerable, but there still exists a need for alternative treatment options to keep fewer patients from needing to receive a subsequent line of therapy.

This study included some limitations that are important to note. First, the possibility that physician responses may have been subject to recall bias. However, the authors note that the physicians were asked to limit their responses to a consideration of only the last 6 months, to help reduce the impact of this bias on study results. Physicians only reported answers based on their own patient pools and practices, thereby limiting the generalizability of the responses. Moreover, as described in the methods, definitions of ORR, PFS, and relapse versus refractory patients were not provided to physicians in the survey questions, and respondent answers were directional estimates based on their own clinical experience and practice. The researchers attempted to distribute physician recruitment in a representative manner, including physicians from major urban hubs across a variety of regions in China; however, due to the focus on urban populations, the survey results may or may not have been reflective of HR+/HER2(−) breast cancer treatment patterns across the larger Chinese population. The authors also note that new agents have been approved in China for HR+/HER2(−) breast cancer since the survey study was conducted (detailed in Supplementary Table 1), including abemaciclib, tucidinostat (chidamide), utidelone, dalpiciclib, and envafolimab [21–23, 25, 26]. The approval of these agents may alter the treatment patterns that were reported by physicians in this study. Regional differences in guidelines, physician preferences, regulatory approvals, and availability of drugs make it challenging to compare and validate treatment patterns in China against that in the US, EU, or Japan. The answers obtained in our survey align closely with national guidelines published at the time that the survey was conducted (September 2019) and shed light on this otherwise little understood but increasingly important market segment. As such, we conduct annual surveys to closely monitor the changes in this vast and rapidly evolving market.

## Conclusions

The survey revealed that physicians in urban mainland China prescribed different treatment modalities and regimens for their HR+/HER2(−) breast cancer patients in 2019. Regimens used in refractory or relapsed patients varied among breast oncologists in China and no apparent standard of care was reported.

## Supplementary Information

Below is the link to the electronic supplementary material.

Supplementary file1 (DOC 57 KB)

## Data Availability

The datasets generated during and/or analyzed during the current study are available from the corresponding author on reasonable request.

## Abbreviations

1L: First line
2L: Second line
3L: Third line
AC: Doxorubicin, cyclophosphamide
CMP: CancerMPact
CSCO: Chinese Society of Clinical Oncology
EC: Epirubicin, cyclophosphamide
EGFR: Epidermal growth factor receptor
EU5: Western Europe
HR: Hormone Receptor
HER2: Human epidermal growth factor receptor 2
NMPA: National Medical Products Administration
NRDL: National Reimbursement Drug List
RWE: Real-world evidence
TCM: Traditional Chinese Medicine
USA: United States
