# Supplementary material for: Treatment of HR+/HER2− breast cancer in urban mainland China: results from the CancerMPact Survey 2019
Source: Breast Cancer Res Treat. 2022 Aug 20;195(3):441–51. doi: 10.1007/s10549-022-06709-x (PMC9464725; doi:10.1007/s10549-022-06709-x)
Supplement: Supplementary file 1 — Supplementary file1 (DOC 57 KB) [file 10549_2022_6709_MOESM1_ESM.doc]

SUPPLEMENTARY INFORMATION

**Treatment of HR+/HER2- Breast Cancer in Urban Mainland China: Results from the CancerMPact Survey 2019**

Journal name: Breast Cancer Research and Treatment

Bhavna Murali, PhD^1^, ORCID: 0000-0003-3546-1416

Laura Durbin, MPH^1^, ORCID: 0000-0002-2766-0899

Sapna Vijaykumar, MBA, PhD^1^

Linda Yang, MBA^1^

Song Li, MBA, PhD^1^

Linda Zhao, MBA, PhD^1^

Stephanie Hawthorne, PhD^1^

Gena Kanas, MPH, PhD^1^

Christine Davis, MS^1^

Otávio Clark, MD, PhD^1^, ORCID: 0000-0003-1607-2589

^1^Cerner Enviza, 2800 Rock Creek Pkwy, North Kansas City, MO 64117, USA

Corresponding Author:

Bhavna Murali

[bhavna.murali@cernerenviza.com](mailto:bhavna.murali@cernerenviza.com)

Tel: +1 614-507-3291

| **Supplementary Table 1. Drugs approved or recommended in China for the treatment of HR+/HER2(-) breast cancer** | | | |
| --- | --- | --- | --- |
| **Agent**  **(generic name)** | **Mechanism of action** | **Setting/Line of therapy** | **CN NMPA Approval** |
| **Agents approved at the time the study was conducted** | | | |
| Avastin®  (bevacizumab) | Anti-VEGF mAb | + paclitaxel  **1L**, metastatic | Available but not approved for breast cancer |
| Abraxane® (albumin-bound paclitaxel;  nab-paclitaxel) | MT binding agent | **1L**, metastatic | June 2008 |
| Afinitor®  (everolimus) | mTOR inhibitor | Advanced HR+/HER2(-) | Available but not approved for breast cancer |
| Caelyx®  (pegylated liposomal  doxorubicin) | Topoisomerase inhibitor | Metastatic breast cancer | Available but not approved for breast cancer |
| Faslodex® (fulvestrant) | Selective estrogen regulator degrader (SERD) | **2L**, HR+ advanced/metastatic | June 2010 |
| Ibrance® (palbociclib) | CDK4/6 inhibitor | + aromatase inhibitor,  **1L**, HR+/HER2(-) | July 2018 |
| Halaven®  (eribulin) | MT-binding agent | **3L**, metastatic | July 2019 |
| **Agents approved after the study was completed** | | | |
| dalpiciclib | CDK4/6 inhibitor | + fulvestrant  **2L+**, HR+/HER2(-) recurrent / metastatic | January 2022 |
| EnWeiDa® (envafolimab) | Anti-PD-L1 mAb | **2L+** (pan-tumor approval), MSI-H/dMMR | November 2021 |
| Epidaza® (tucidinostat; formerly chidamide) | HDAC inhibitor | + aromatase inhibitor  **2L**, HR+/HER2(-) metastatic | November 2019 |
| UTD (utidelone) | MT inhibitor | + capecitabine  **2L+**, recurrent / metastatic | March 2021 |
| Verzenios® (abemaciclib) | CDK4/6 inhibitor | + fulvestrant  **2L**, HR+/HER2(-) advanced / metastatic | December 2020 |
|  |  | + aromatase inhibitor  **1L**, HR+/HER2(-) advanced / metastatic | December 2020 |
|  |  | + tamoxifen / aromatase inhibitor,  **Adjuvant**, HR+/HER2(-) Ki-67 ≥20% | January 2022 |

**Abbreviations**: 1L = First-line, 2L = Second-line, 3L = Third-line
